# A path-based analysis of infected cell line and COVID-19 patient transcriptome reveals novel potential targets and drugs against SARS-CoV-2

**DOI:** 10.21203/rs.3.rs-1474136/v1

**Published:** 2022-03-21

**Authors:** Piyush Agrawal, Narmada Sambaturu, Gulden Olgun, Sridhar Hannenhalli

**Affiliations:** 1 Cancer Data Science Laboratory, National Cancer Institute, National Institutes of Health, Bethesda, MD, USA; 2 Theoretical Biology and Biophysics, Los Alamos National Laboratory

**Keywords:** SARS-CoV-2, Covid-19, Differentially Expressed Genes, Network Based Approach, Therapeutic Target, Drugs

## Abstract

Most transcriptomic studies of SARS-CoV-2 infection have focused on differentially expressed genes, which do not necessarily reveal the genes mediating the transcriptomic changes. In contrast, exploiting curated biological network, our PathExt tool identifies central genes from the differentially active paths mediating global transcriptomic response. Here we apply PathExt to multiple cell line infection models of SARS-CoV-2 and other viruses, as well as to COVID-19 patient-derived PBMCs. The central genes mediating SARS-CoV-2 response in cell lines were uniquely enriched for ATP metabolic process, G1/S transition, leukocyte activation and migration. In contrast, PBMC response reveals dysregulated cell-cycle processes. In PBMC, the most frequently central genes are associated with COVID-19 severity. Importantly, relative to differential genes, PathExt-identified genes show greater concordance with several benchmark anti-COVID-19 target gene sets. We propose six novel anti-SARS-CoV-2 targets ADCY2, ADSL, OCRL, TIAM1, PBK, and BUB1, and potential drugs targeting these genes, such as Bemcentinib, Phthalocyanine, and Conivaptan.

## Introduction

COVID-19, a serious respiratory disease caused by the zoonotic virus Severe Acute Respiratory Syndrome Coronavirus 2 (SARS-CoV-2, or SC2 for short), has emerged as a global pandemic leading to ~315 million infections and 5 million deaths (data till date January 10^th^, 2022, as per WHO dashboard). Despite a huge body of research investigating the SC2 biology (1), host-virus mechanisms (2), potential drug targets and their inhibitors (3), we are far from a complete understanding of mechanisms underlying the varied COVID-19 symptoms, and the search for an effective therapy continues. As there are limited treatment options available for SC2, several drugs which are prescribed to treat infections by other viruses such as Middle East respiratory syndrome (MERS), severe acute respiratory syndrome (SARS), human immunodeficiency virus (HIV), etc. have been tried on COVID-19 patients (4). Remdesivir, a viral RNA dependent RNA polymerase inhibitor used in the treatment of Ebola virus, was the first FDA-approved drug for the treatment of COVID-19 (5); however, it has not been broadly effective. More recently approved Paxlovid was found to be effective against multiple SC2 variants including Omicron (6). While several effective vaccines are now in general use, there continues to be an urgent need for more effective therapeutic options for infected patients, especially considering the highly varied, and sometimes long-term effects as well as side effects of the current therapies.

Viruses hijack the host system for their own survival and proliferation, especially by exploiting and manipulating host transcriptional machinery and gene regulation (7). A better understanding of the host transcriptomic response to the viral infection is thus widely recognized as critical in designing better therapeutics strategies (4). Most previous studies investigating infection-induced transcriptomic changes in the host tissues and immune cells focus on genes that are differentially expressed (DEGs) upon infection and perform a series of downstream analyses to decipher the underlying mechanisms (8). One issue with the DEG-centric approach is that certain genes are known to be differentially expressed in a wide variety of contexts and represent generic transcriptional response and are not specific to SC2 infection (9). Moreover, it is now well recognized that differential expression ought to be interpreted in the context of genetic networks and pathways, and indeed some of the works have investigated SC2 transcriptomic response data from a network perspective (10, 11). These approaches nevertheless rely on gene-level differential expression as the lynchpin for the downstream network-assisted analyses. An alternative approach - PathExt - that we have recently shown to be superior to DEG-centric approaches (12), instead integrates transcriptomic data with curated gene networks and instead of identifying differentially expressed genes, identifies differentially active paths in the integrated network, and then identifies the central genes mediating the differential activities of the most perturbed paths. This alternative approach is based on the recognition that (i) gene expression is noisy and DEGs can therefore lead to false positives, and (ii) key regulatory genes that mediate global transcriptomic changes and thus present a potent target may themselves not be differentially regulated and will thus be missed by DEG-centric approaches.

Here, using PathExt, we comprehensively analyze and compare transcriptomic response to SC2 and other respiratory viruses (SARS-CoV-1, MERS, Influenza, RSV, and HPIV3) in multiple cell lines (A549, A549-ACE2, Calu3, Vero, MRC5 and NHBE), as well as in COVID-19 patient-derived peripheral blood mononuclear cells (PBMCs). While PathExt identifies largely distinct sets of central genes across cell lines and viruses, these genes nevertheless converge on common processes such as cytokine signaling, cell cycle, metabolism, etc.; however, we observe a much greater similarity in response across cell lines for the same virus than across viruses in the same cell line. We assess the complementarity and unique advantages of using PathExt compared to the conventional DEG-based approach and find that PathExt genes capture experimentally identified anti-SC2 targets more accurately than DEGs, while also providing an overall greater enrichment of key biological processes. In PBMC data, we find that PathExt-identified central genes are associated with patient severity. Finally, we propose novel anti-SC2 therapeutic targets and their potential inhibitors that are either FDA-approved or currently in clinical trials.

## Results

### Overview of the approach

**Figure 1:**
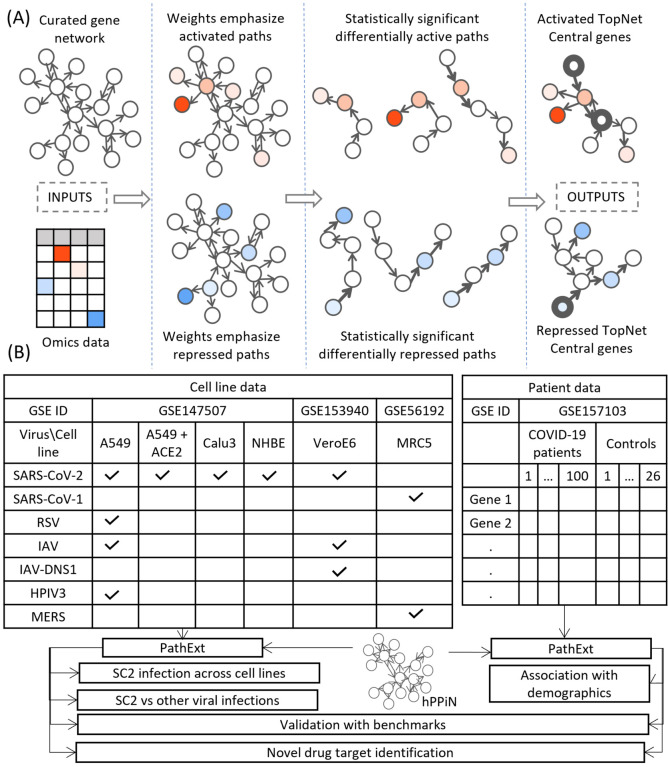
Study Workflow. **(A)** Our PathExt tool accepts as input a curated gene network and gene expression data, to output two weighted sub-networks - an activated and a repressed TopNet comprising activated and repressed paths respectively, and a list of central genes in each TopNet. Activated genes are shown here in shades of red, and repressed genes are in shades of blue. PathExt integrates the inputs such that edges connecting genes with substantial change in expression are preferentially traversed by a shortest paths algorithm (Methods), illustrated here using wider arrows. Shortest paths which are statistically significant (permutation based) now represent differentially active (or repressed) paths and make up the TopNets in which PathExt identifies central genes based on ripple centrality. **(B)** We apply PathExt to analyze RNa-seq data from SARS-CoV-2 infection in both cell lines and patient PBMC data. PathExt outputs from the cell line data are used to compare cross-cell-line variation in SC2 infection response, and within-cell-line variation in response to other viruses. In patient PBMC data, we identify associations between PathExt results and demographics. We then validate all the results against benchmarks and use the TopNets and central genes to propose novel drug targets, as well as novel drugs for known targets.

PathExt is our recently published tool; here we provide a brief intuitive overview of the approach. The aim of PathExt is to identify differentially active paths, in a prior knowledge-based gene network, while comparing transcriptomic data in two conditions, and shortlist genes among those paths which might be critically mediating the observed phenotypic change. As noted above, in contrast to conventional DEG-centered approaches, such mediating genes may not be differentially expressed themselves. The PathExt workflow is illustrated in Fig. 1A. PathExt starts by integrating a knowledge-based curated gene network with sample-specific omics data from the conditions of interest; we employed a curated network - HPPIN - which integrates physical, regulatory, and metabolic interactions between genes or proteins (13). In each sample separately, nodes and edges of the network are weighted such that interactions involving differentially expressed genes are preferentially traversed by a shortest path algorithm. This integration is done in two ways, to emphasize either differential activation or repression. Shortest paths whose weights are statistically significant (based on permutation) can then be interpreted as statistically significant differentially active (or repressed) paths. Such paths are then cast as a sub-network referred to as the TopNet, either activated or repressed, based on the weighting scheme. PathExt then identifies central genes in each TopNet based on ripple centrality (14), which captures genes that can reach a large part of the TopNet along highly active (or repressed) paths. TopNet and the central genes are identified independently for each transcriptomic sample.

Here, we apply PathExt to analyze how the impact of SC2 infection (as well as other respiratory viruses) can vary in different cell lines, as well as in patient derived PBMCs (Fig. 1B). We first compare SC2 infection across cell lines, and with other viral infections in the same cell lines. In PBMC data, we identify associations between PathExt results and patient demographics. We then show a high concordance between the results from both cell line and PBMC data, and benchmarks such as genes previously shown to be affected by SC2 infection as well as experimentally screened drugs. Based on this, we use the output of PathExt to propose novel drug targets as well as drugs against them.

### Key processes mediating SC2 infection across cell lines

**Figure 2:**
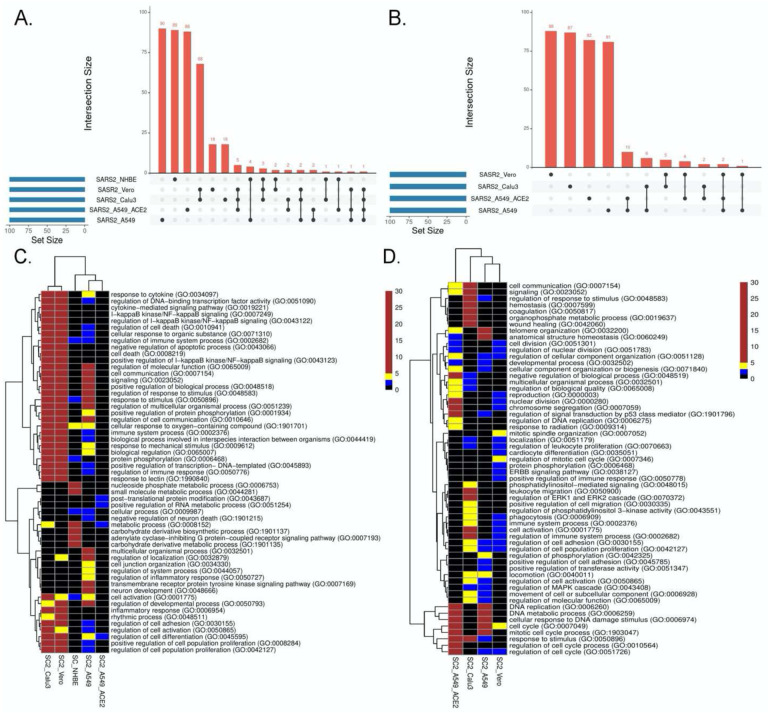
SC2 infection across cell lines. We analyzed the transcriptional response across various cell lines post SC2 infection. We obtained the top100 central genes from each cell line for both TopNets (activated & repressed) and compared the gene commonality across cell lines as shown in upset plots for activated TopNets (Fig. 2A) and repressed TopNets (Fig. 2B). Next, we performed gene enrichment analysis and compared the top 10 parent GO terms enriched in various cell lines for activated TopNets (Fig. 2C) and repressed TopNets (Fig. 2D). Here we show only those GO terms that were significant in at least 2 cell lines. Complete list of enriched GO terms is provided in the supplementary tables. Heatmap is created by converting the FDR corrected p-value of each GO terms to −log10 scale. Significance of the terms is shown in various color ranges (0–1.3, 1.3–2.0, 2–5 and >5–30). All the non-significant processes are shown in black (value <1.3).

We compared the transcriptional responses between five cell lines infected by SC2. These included three lung epithelium derived cell lines -- A549, A549 with increased ACE2 expression (A549-ACE2), and Calu3, Vero cell line derived from African monkey kidney, and primary bronchial epithelium cell line – NHBE (15, 16). In each case, we applied PathExt to identify the top 100 central genes each in the activated and repressed TopNets (Supplementary Table S1); no repressed TopNet was detected in NHBE. We found that the top 100 genes were largely disjoint across cell lines (Fig. 2A and 2B) with one exception, where the top 100 genes from the activated TopNets in Calu3 and Vero cell lines shared 79 common genes; indicating a high cell-type specificity in response to SC2 infection. A list of the top 100 upregulated and downregulated DEGs is provided in the Supplementary Table S2.

We then performed functional enrichment on the top 100 genes identified from each dataset using PANTHER (17), and consolidated (Methods) the enriched biological process terms using REVIGO (18). In case of activated TopNets (Fig. 2C), as expected, Calu3 and Vero cells show similar upregulated pathways such as I-kappaB kinase/NF-kappaB signaling, regulation of transcription factor (TF) activity, cytokine mediated signaling pathway, and response to stimulus. These processes are well supported by previous studies. Regulation of immune signaling pathways is another process prominently observed in COVID-19, especially production of various cytokines and chemokines (19, 20). Likewise, hyperactivation of NF-kappaB signaling pathway post SARS-CoV-2 infection has been observed (21) and NF-kappaB has been recognized as a potential pharmacological target to treat COVID-19 (22). Several host TF binding sites (TFBS) are present in SARS-CoV-2 genome which the virus exploits for its replication (23). Calu3, Vero, and A549 were enriched for inflammatory response, specifically, tyrosine kinase signaling, recapitulating previously established links between SARS-CoV-2 and tyrosine kinase signaling (24, 25). In A549 cells with high *ACE2* expression, the strongest enrichment was seen for the post-translational protein modification process. Although inflammatory and immune response pathways were not statistically enriched among the top 100 genes in A549-ACE2 cell line, key immune response genes were among the top 100 central genes, e.g., *STAT3, STAT5A, NFKB1, NFKBIA*, etc.

Other enriched processes such as metabolism and cell differentiation also have known roles in viral infections. These results suggest that the host response to viral infection may depend on the infection and replication rates in various cell lines. For instance, the differential response in A549 may be because A549 cells are relatively less permissive to SARS-CoV-2 replication owing to lower concentration of *ACE2* receptor protein required for viral entry (26), while Calu3 and Vero cell lines exhibit a much higher expression of *ACE2*. This difference in infection rate leads to differences in the host immune responses, which is consistent with previous studies (16, 27). Key genes in NHBE (bronchial epithelium cell line) were strongly associated with small molecule and nucleoside phosphate metabolic processes, known to be central for viral replication and survival (28). A complete list of all the upregulated enriched terms as well as the parent-child relations identified for various SARS-CoV-2 infected cell lines is provided in Supplementary Table S3.

In addition to biological process, we also looked at the molecular function associated with the top 100 central genes present in the Activated TopNets across these cell lines (Supplementary Table S4). In the case of A549, we saw the enrichment of molecular functions associated with DNA-binding transcription factor activity, calcium-dependent protein kinase C activity, protein binding, etc. All these processes have been shown to be important for SC2 infection. For instance, the complex of viral S protein and host *ACE2* receptor binds to calcium ions (EF-hand domain) and shows protein kinase activity (29). After binding, the spike protein is further cleaved by *TMPRSS2*, a transmembrane protein serine 2 leading to further downstream signaling processes (30, 31). As observed with biological processes, molecular functions for Vero and Calu3 cell lines were very similar. They were enriched for the functions such as protein kinase activity, TNF receptor superfamily binding, cytokine receptor binding, etc. These findings are supported by previous published literatures (32, 33). Lastly, for NHBE cell line, we saw molecular functions such as small molecule binding, carbohydrate derivative binding, etc. as shown in previous works (34, 35). In stark contrast, for top 100 DEGs, only one statistically significant molecular function was enriched across all cell lines, namely, “sequence specific DNA binding function” in the Vero cell line. The enrichment of regulatory and signaling molecules among the PathExt genes underscores the rationale that PathExt attempts to identify key genes mediating the transcriptional response.

Fig. 2D summarizes the pathways enriched among the top 100 central genes in the repressed TopNets. A549 cell lines were highly enriched in cell cycle, DNA replication, and DNA damage response; interestingly, the enrichment was much greater in A549 with *ACE2*. These observations recapitulate the established biology of viral infection. For instance, genes mediating DNA damage response -- *ATM* and ATM/Rad3-related (*ATR*) -- were among the top 100 central genes. Key genes in Vero cells are enriched for mitotic spindle organization, ERBB signaling, EGFR signaling, protein phosphorylation, phagocytosis, etc., all previously known to modulate SARS-CoV-2 infection response (36, 37). Lastly, in Calu3 cells, we saw enrichment of several lipid metabolism pathways. Lipids are one of the major components of viral structure and play an important role in its entry and replication inside the host cell. Several studies have shown the potential role of lipids and lipid induced metabolic changes in coronavirus infection (38, 39). Other enriched pathways include leukocyte migration and phagocytosis. The downregulated enriched biological terms identified for various SC2 infected cell lines is provided in Supplementary Table S5.

We also performed molecular function analysis for the top 100 central genes in repressed TopNets (Supplementary Table S6). For A549 cell line, functions such as catalytic activity and single-strand DNA binding, were enriched. These processes are associated with SC2 attachment, infection and downstream signaling events as shown in various studies (40, 41). Likewise, for A549 cell line with high ACE2 expression, “single-stranded DNA binding” was enriched. Genes associated with this function (e.g., *ATM* and *ATR*) are essential for response to DNA damage and repair, DNA metabolism, and maintaining genomic stability (42, 43). In case of Calu3 cell lines, enriched functions included kinase activity, purine ribonuclease triphosphate binding, signaling receptor binding, etc. There are various reports which explain how these functions plays an important role in viral entry, membrane trafficking, signaling, etc. (32, 44).

We investigated the commonality between the activated and repressed TopNets for the cell lines. We first confirmed that the top 100 central genes of the activated and repressed TopNets of the same cell line were disjoint, with a few exceptions: out of 100 genes, 1 is common in Calu3 cell line, 2 in A549, 3 in A549 with high *ACE2* expression, and 5 in the Vero cell line, suggesting dual activation and repressive roles of certain key genes; for instance, *ATM*, a protein kinase which plays key roles in many processes such as cell cycle progression, cell metabolism and growth, oxidative stress and chromatin remodeling, and is upregulated as well as downregulated in different cancers (45). Indeed, *ATM* is known to regulate these pathways in COVID-19, as shown in previous study (46). Despite disjoint central genes between activated and repressed TopNets, the enriched pathways among the central genes show greater commonality, such as protein phosphorylation, suggesting that these key processes may be involved in both activation and repression of different downstream processes.

### Comparison of SARS-CoV-2 infection with other viral infections

**Figure 3:**
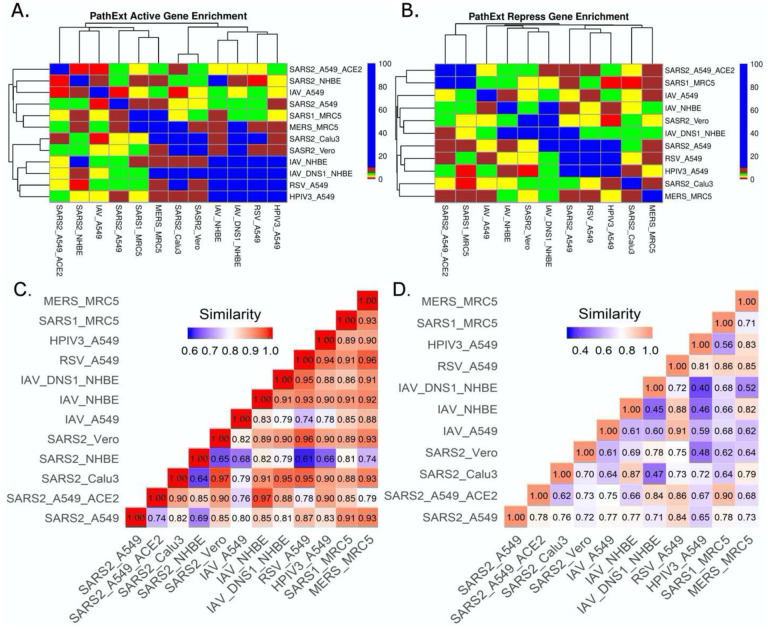
SC2 infection comparison with other viruses. We analyzed the transcriptional response to different viruses including SC2 in various cell lines. We obtained the top 100 central genes from each cell line for activated & repressed TopNets and compared the gene commonality across various viruses in different cell lines as shown in gene enrichment plot (Observed/Expected overlap) for activated TopNets (Fig. 3A) and repressed TopNets (Fig. 3B). Semantic similarity among the enriched biological processes observed in different viruses activated and repressed networks (Fig. 3C&D respectively) shows higher similarity.

Next, we compared the host transcriptional response to SC2 infection with those for other respiratory viruses in Calu3, Vero, MRC5, NHBE and A549 cell lines where there was data for an additional respiratory virus (RSV, IAV, HPIV3, SARS-CoV-1 and MERS). Top 100 central genes from each viral TopNet (activated & repressed) were used for comparison. As shown in Fig. 3A&B, while there are small but significant overlaps in most cases, the responses could be considered largely virus specific.

Amongst activated TopNets, the SC2 response shares at least 10 central genes with responses to influenza virus, RSV, SARS-CoV-1 and MERS virus in different cell lines. SC2 shared 29 genes with MERS in the MRC5 cell line. Influenza virus in NHBE (bronchial epithelium) lacking NS1 shared 27 genes with SC2-infected Calu3 (Lung epithelium) and 25 genes with SC2-infected Vero cell line. The commonly shared genes between SARS-CoV-2 and most of the other viruses included *MAPK1, IL7, LYN, STAT3, TRAF6, NFKB1*. These genes are associated with pathways such as regulation of MAPK, response to cytokine, positive regulation of cell differentiation, and innate and adaptive immune response, all of which are supported by previous experimental work (16, 33). These overlaps among the top 100 genes are significant and suggest shared responses across viruses. In repressed TopNets, a similar overlap trend was seen. SC2 shares at least 10 genes with all the viruses in different cell lines except MERS. Among the top 100 central genes in the repressed TopNet, SC2 shares 13 common genes with RSV and HPIV3 in the A549 cell line. Some of the commonly shared genes between SC2 and other viruses included *E2F1, BRCA1, RAD21, CDK, DDAH2*. These genes are associated with pathways such as cell cycle process, apoptotic process, cellular nitrogen compound metabolic process, DNA repair, etc., again revealing known shared responses across viruses (16, 47).

Next, we assessed the similarity in responses between SC2 and other viruses at the pathway level, using PANTHER and REVIGO. Given the enriched terms in the TopNets for all the viruses, we computed the semantic similarity using the GOSemSim package in R (48). For the activated TopNet, SC2 shares high similarity with nearly all the viruses considered in the study (Fig. 3C). Highest similarity of 0.97 is observed between the processes enriched in SC2-infected A549 with high ACE2 expression, and Influenza-infected NHBE. Activated TopNets across viruses share cytokine signaling, NF-kappaB signaling, inflammatory response, DNA binding transcription factor activity, and protein phosphorylation, all of which are well established host responses to respiratory viral infections. The processes which were uniquely enriched for SC2 infection include ATP metabolic process, G1/S transition of mitotic cell cycle, post-translational protein modification, and carbohydrate derivative metabolic process. Some recent published studies also account for these processes associated with SC2 infection (46, 49, 50). A complete list of enriched biological pathways in activated TopNets for SC2 and other viruses is provided in the Supplementary Table S3.

Compared to activated TopNets, repressed TopNets exhibited greater divergence in terms of enriched processes (Fig. 3D), but consistent with activated TopNets, we observed a greater similarity across different cell lines infected by SARS-CoV-2. Key enriched processes across viruses include DNA replication, telomere organization, DNA metabolic process, cellular response to DNA damage stimulus, regulation of MAPK cascade, negative regulation of apoptotic process, regulation of cell migration, nuclear division, regulation of cell cycle, etc., all of which have literature support (51, 52). However, the unique processes enriched with SC2 infection include activation of phospholipase C activity, cardiomyocyte differentiation, leukocyte activation and migration, and phagocytosis. Complete list of the enriched biological pathways in repressed TopNets for SC2 and other viruses is provided in the Supplementary Table S5. Overall, while as expected the functional response to SC2 infection is similar with the response to other respiratory viruses, our analysis reveals unique aspects of SC2 response. Functional relevance of the unique processes enriched among central genes in host response to SC2 response, however, will require further experimental follow up.

### PathExt provides unique insights compared with DEG analyses

**Figure 4:**
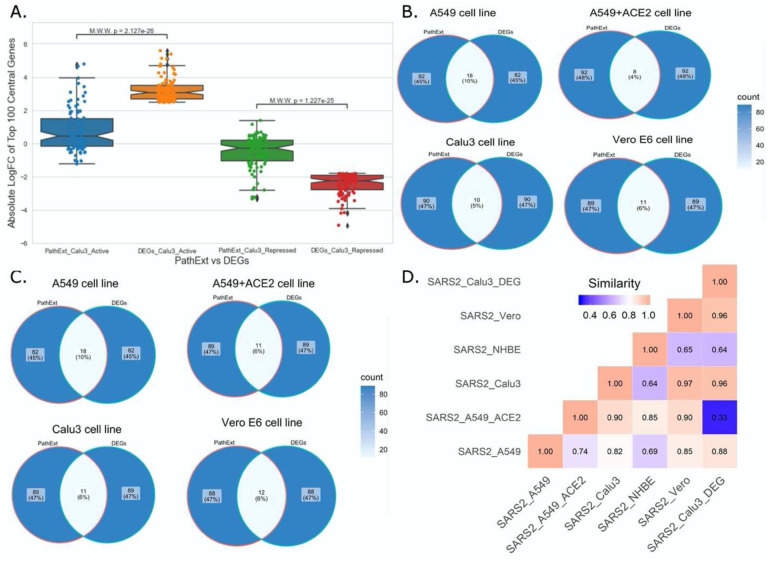
Comparison of PathExt central genes with DEGs. We compare the PathExt result with the results obtained using traditional DEG approach. (A) LogFC comparison of the top 100 genes between PathExt and DEGs obtained from the SC2-infected Calu3 cell line. Differential expression is estimated in infected relative to uninfected cells. (B, C) Venn diagram shows the gene overlap among the top100 central genes & DEGs present in activated (B) network and repressed (C) network across different cell lines. No repressed TopNet was seen in NHBE. (D) Semantic similarity among the enriched PathExt and DEGs biological processes for activated TopNets.

Next, to assess advantage or complementarity of PathExt relative to DEG-centric approach, we compared the genes and pathways identified by PathExt with DEGs and their enriched pathways. Recall that PathExt identifies central genes that potentially mediate differential expression of other genes but may not be differentially expressed themselves. To test this, we selected the SC2-infected Calu3 cell line data and analyzed the log-fold change in expression of the top 100 central genes identified by PathExt TopNets and DEGs. As shown in Fig. 4A, log-fold change of the genes present in the PathExt TopNets are much smaller than those of the DEGs, and therefore would systematically go undetected in a differential expression analysis. Similar results was seen for other SC2 infected cell lines as well (Supplementary Fig. S1).

Next, in each SC2-infected cell line, top 100 upregulated genes were compared with the top 100 central genes in activated TopNet, and top 100 downregulated genes were compared with the top 100 central genes in repressed TopNet. As shown in Fig. 4B and 4C, PathExt genes and DEGs were largely distinct. Given minimal overlap between PathExt central genes and DEGs, we compared them in terms of enriched processes. Remarkably, in stark contrast with PathExt central genes, the 100 most downregulated DEGs do not reveal any pathway enrichment in any of the cell lines, and the upregulated DEGs showed pathway enrichment only in Calu3 and Vero cell lines (Supplementary Table S7); in Vero cell line only one process was enriched and is therefore not discussed. As expected, pathways enriched among upregulated DEGs in Calu3 showed highest similarity with those in activated TopNet in Calu3 (Fig. 4D), despite very little overlap in terms of genes. In Calu3, while the common pathways among PathExt and DEGs (Supplementary Fig. S2) includes response to cytokine production, and immune system process, several pathways were uniquely revealed by PathExt, including regulation of DNA binding transcription factor activity, I-kappaB kinase/NF-kappaB signaling, regulation of cell death, and cellular response to organic substances. These pathways are well-associated with the COVID-19 infection as shown in multiple studies (19, 23, 53), highlighting the relative advantage of PathExt over the conventional DEG approach.

### Application of PathExt to COVID-19 patient PBMC

**Figure 5:**
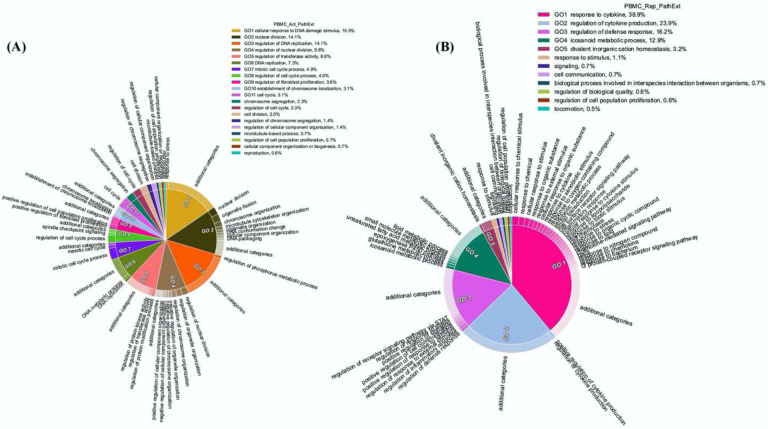
Functions enriched among central TopNet genes in patient PBMCs. Most frequent top 100 central genes were obtained from the activated and repressed networks across patient PBMC data. Enriched biological processes were obtained by performing Gene Ontology study followed by parent child relationship, shown in the form of circular visualization plot for activated TopNet (A) and repressed TopNet (B).

Next, we applied PathExt to 100 COVID-19 positive and 26 negative control individuals’ PBMC transcriptomic data from (54), revealing activated TopNets in 96 samples and repressed TopNets in all 100 samples. As above, we first identified the top 100 most central genes in each TopNet, and then integrating across samples, we obtained the top 100 most frequent central genes separately for the activated and the repressed TopNets. Genes *PBK, CDC6*, and *BUB1*, were found to be the most frequent central genes among the activated TopNets occurring in 56%, 53%, and 48% of the 96 samples, respectively. Likewise, in the repressed TopNets, IL6 was the most frequent, occurring in 49% of the samples, followed by *POMC* in 48% of the 100 samples; a complete list of top genes is provided in Supplementary Table S8. As shown in Fig. 5A, pathways enriched among the frequent central genes in activated TopNets are predominantly related to cell cycle, which is an expected response to infection by the host immune system (55), as well as potentially linking SC2 infection with cancer (56, 57). Previous studies have observed that cancer patients are more vulnerable to SC2 infection leading to adverse outcomes, likely due to compromised immune system (58). Some of the pathways such as uncontrolled production of cytokines, type-I interferon (*IFN*-*I*), dysregulation of immune checkpoint signaling, etc. are common in both COVID-19 and cancer. Complete list of enriched terms associated with top 100 most frequent central genes in the activated TopNets are provided in the Supplementary Table S9. Molecular function enrichment analysis revealed cell cycle and replication processes such as single-stranded DNA binding, cyclin dependent protein serine/threonine kinase regulator activity, etc. These are well known functions which occur post SC2 infection (4, 59). Interestingly, top 100 upregulated DEGs show only one molecular function enriched, namely, 2’−5’-oligonucleotide synthetase activity (Supplementary Table S10).

In contrast, pathways enriched among the frequent central genes in the repressed TopNets included response to cytokine, regulation of cytokine production, regulation of defense response and icosanoid metabolic process (Fig. 5B). Suppression of regulatory mechanisms that keep cytokine production in check is consistent with observed cytokine storms in COVID-19 patients and ensuing damage of organs such as the liver leading to downregulation of various metabolic processes (60, 61). Likewise, SC2 can suppress the host immune defense by downregulating the T-cell function (62), cytokine production and cell-cell adhesion (60, 63). Complete list of enriched terms associated with the top 100 most frequent central gene is provided in the Supplementary Table S11. Molecular functions associated with top 100 central genes include receptor ligand activity, heme binding, chemokine binding, etc. Once again, these are well known functions which occur post SC2 infection (64, 65). However, downregulated DEGs were found to be enriched only for retinol binding function. Complete list of molecular function associated with repressed TopNet in PBMC is provided in Supplementary Table S12.

The cellular response in COVID-19 patient PBMC likely reflects immune response to systemic infection by SC2. However, there is some evidence that SC2 can infect immune cells as well (66–68), and therefore, the cellular response in PBMC could in part reflect endogenous response by the infected PBMC cells. To distinguish between these two possibilities, we identified the enriched pathways separately for the PBMC central genes shared with those in other cell lines (reflecting endogenous response to infection) and the ones unique to PBMC (potentially reflecting immune response). For activated TopNet, we therefore compared the 1,830 unique genes appearing among the top 100 central genes in any TopNet across patient PBMC samples with the 395 unique genes among the 100 central genes across 5 cell lines. The 156 common genes were enriched in the similar pathways as observed in cell lines, such as regulation of DNA-binding transcription factor activity, regulation of I-kappaB kinase/NF-kappaB signaling, etc. The remaining PBMC-specific 1,674 genes were enriched for the pathways such as response to cytokine, humoral immune response, cellular response interleukin-4, and leukocyte proliferation, representing immune response to systemic infection. Likewise, for the repressed TopNet, we compared 1,877 unique genes in PBMC TopNets with the 368 genes across cell lines. The 148 common genes were enriched for the pathways such as positive regulation of cell migration, response to DNA damage stimulus, phosphatidylinositol metabolic process, etc. The remaining PBMC-specific 1,729 genes were enriched for T-helper-2 cell differentiation, cellular response to interleukin-18, positive regulation of MHC class II biosynthetic process, regulation of eosinophil migration, regulation of neutrophil mediated cytotoxicity, suggesting an overall suppression of immune response to systemic infection. These results are consistent with previous studies (69, 70). Zhou et.al. have shown that an individual who has gone through COVID-19 and has started testing SC2-negative may still exhibit cold symptoms due to decreased expression of adaptive immune related genes, especially those related to T and B cells and HLA molecules, making them susceptible to secondary infections. They also showed that the suppression of the adaptive immune system could be due to dysregulated host response and not because of immune checkpoint molecules such as PD-1, PD-L1, CTLA4, etc. (71). Overall, PathExt reveals immune cell response to SC2 infection and discriminates, to some extent, the potential cell-endogenous responses by infected PBMC and response to systemic infection. All enriched GO processes for activated and repressed TopNets in patient PBMCs common with cell lines are provided in Supplementary Table S13 whereas enriched GO processes for activated and repressed TopNets unique to patient PBMCs are provided in Supplementary Table S14.

**Figure 6:**
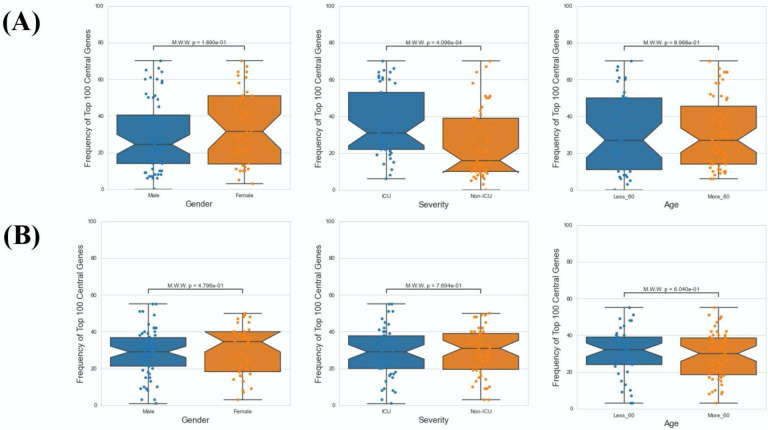
Demographic features analysis. Mann Whitney Test was performed to check statistical significance between top 100 central genes and various demographic features (age, sex and severity). In case of PathExt identified top genes, “Severity” was found to be the only statistically group among activated TopNet genes (A). However, no group was statistically significant in case of repressed TopNet (B).

Next, we investigated the association between PathExt-identified most frequent central genes in PBMCs and the available demographics and clinical features of the patients -- age (< = 60 years or >60 years), sex (male vs female), and severity (ICU vs non-ICU). We found that most frequent genes in activated TopNets were more frequently observed in severe cases (Fig. 6A). We did not see a direct association between frequent central genes and sex or age. Frequency and distribution of the PathExt identified genes in patients with severe illness is consistent with previous findings (54, 72). To further probe into association of frequent central genes with severity, we identified genes that were uniquely central in either ICU or non-ICU patients, with a minimum frequency of 5 (Supplementary Table S15). Central genes in ICU patients included genes like *CDC25C, LOX, TGFB3, CCNF*, etc. and were enriched for mitotic cell cycle phase transition and regulation of cell death. Central genes in non-ICU patients included genes such as *CCR10, IRF6, CCL4*, etc. and were enriched for cytokine-mediated signaling pathway, cell communication, positive regulation of ERK1 and ERK2 cascade (Supplementary Table S16).

The analysis above identifies frequent central genes in ICU and non-ICU patients. We further aimed to assess whether the TopNet neighbors of the central genes are similar across samples, which would indicate a homogeneity in the perturbed paths mediated by a central gene. Toward this, we quantified, for each central gene independently, all sample-pair overlap (quantified by Jaccard Index or JI) between the sample-specific TopNet neighbors of the central gene. We did this separately for ICU and non-ICU patients; Supplementary Figure S3 shows the distribution of JIs for the central genes in ICU and non-ICU patients separately, suggesting a greater homogeneity of response mediated by the central genes in the ICU patients.

We did not notice any association between frequent genes in the repressed TopNets and demographic features (Fig. 6B). For DEGs, top central genes were more associated with male in activated TopNets and with severe cases in repressed TopNets (Supplementary Fig. S4)

As a point of comparison, we also identified the upregulated and downregulated DEGs from the COVID-19 patients. As above, we selected the top 100 upregulated and downregulated genes in each patient sample and then the top 100 most frequent upregulated and downregulated genes across all 100 patients. First, we note that while frequent central genes in activated TopNets exhibit slightly lower fold changes compared to frequent DEGs, the frequent central genes in repressed TopNets exhibit far lower fold changes compared to DEGs (Supplementary Fig. S5).

Next, we analyzed the commonality among the PathExt and DEGs top 100 genes. We found 28 genes to be common among the activated TopNets and upregulated DEGs and 8 were common among the repressed TopNets and downregulated DEGs. Next, we analyzed the similarity at the pathway level. While many processes enriched among frequent central genes were also enriched among upregulated DEGs (e.g., cell cycle) (Supplementary Fig. S6), the PathExt central genes uniquely revealed cellular response to DNA damage stimulus, regulation of transferase activity, regulation of fibroblast proliferation, establishment of chromosome localization, etc. However, the frequent downregulated DEGs did not identify any significant enriched process, underscoring the relative advantage of the PathExt approach.

### PathExt reveals previously ascertained anti-SC2 target genes far better than DEGs

**Figure 7:**
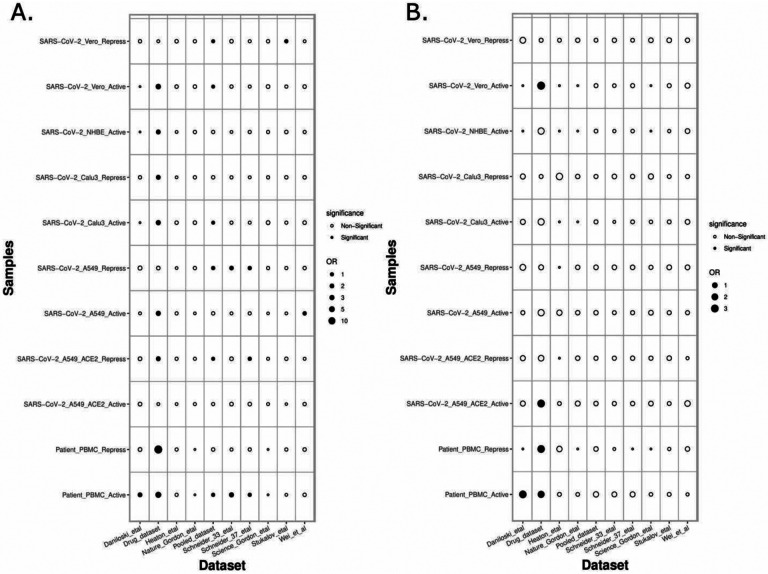
Overlap of PathExt-identified genes and DEGs with previously published datasets. Fig. 7A the overlap of the PathExt identified top 100 activated & repressed genes of various cell lines and PBMC datasets with various previously published drug validation datasets (CRISPR-Cas, Host-Virus PPI networks, Drug-target studies). Fig. 7B the overlap of the top 100 upregulated & downregulated DEGs of various cell lines and PBMC datasets with the same drug validation datasets.

Next, we assessed the extent to which the PathExt-identified genes and DEGs recapitulate previously proposed anti-SC2 target genes based on experimental screens. Toward this, we compared the top 100 central genes identified in SC2-infected cell lines and patient PBMC data against previously published benchmark datasets of anti-SC2 targets. In total, we compiled 11 gene sets from our study: 6 for activated TopNets (5 for the cell lines and 1 for the PBMC cohort) and 5 for repressed TopNets (4 for the cells lines and 1 for the PBMC cohort); while for each cell line we considered the top central genes, for PBMC, we selected the 100 most frequent central genes across patients. We compiled 9 benchmark genes sets from previously published reports which includes CRISPR gene-knockout studies (73–76), viral-host protein-protein interactions (PPI) (77–79) and targets associated with experimentally screened drugs in various cell lines and animal models (76, 80–82) (Methods). Next, we assessed overlap between our 11 gene sets and the 9 different benchmark datasets using Fisher’s Exact test, resulting in 99 tests. In 18 of the 99 comparisons (expectation is ~5 at p-value threshold of 0.05) PathExt gene sets significantly overlapped with the benchmark gene sets (Fig. 7A). In sharp contrast, analogous sets of DEGs significantly overlapped with gold sets in only 5 cases, as expected by random chance (Fig. 7B), again underscoring the relative advantage of PathExt.

### Identifying novel potential anti-SC2 targets and drugs

Next, to identify novel drug targets against SC2, we removed the already known targets (Methods) against SC2 and other viruses (considered in this study) from the TopNets (activated & repressed). Based on the frequency of these unique genes in the TopNets of various SC2 infected cell lines, we proposed novel anti-SC2 targets namely *ADCY2, ADSL* (mediating activated TopNet), and *OCRL*, and *TIAM1* (mediating repressed TopNet). Similar analysis performed for the PBMC data reveals that genes like *PBK* and *BUB1* can be potential new targets. Complete list of the potential targets observed in both cell line and PBMC is provided in the Supplementary Table S17. The gold standard dataset used to ascertain novel targets was created in March 2021. Instead of using an updated dataset, we decided to further assess the accuracy of our proposed novel targets in a prospective manner: a quick survey of the literature published since March 2021 validated some of the targets that we have identified, including *ESRRA* (83), *FBXW11* (84), *PTGDR2* (85), *EGFR* (83)*, CTCF* (86), *CYP2E1* (87), and *PPARG* (88). This serves as a prospective validation of the targets proposed by PathExt. Finally, we performed virtual screening (Methods) to identify potential inhibitors against the proposed novel targets in our study. We propose the top 5 potential drug molecules for each target (cell line and PBMC) in Table 1.

**Figure 8:**
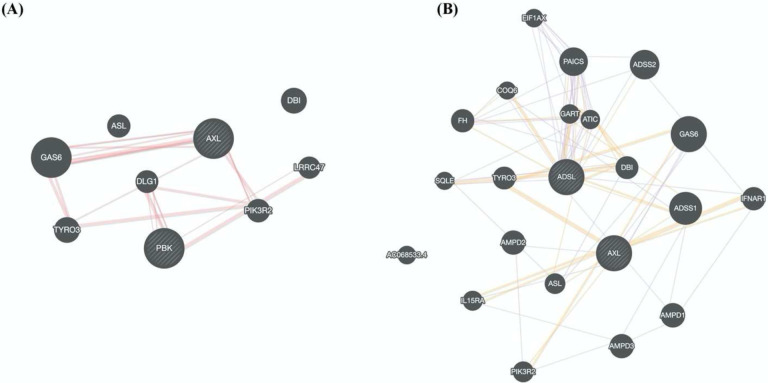
Drug-target association. Based on our virtual screening study, we identified, Bemcentinib as a potential inhibitor for PBK and ADSL. Bemcentinib is a well-known drug against AXL, and we saw that this gene (AXL) is connected to our proposed target PBK (Fig. 8A) and ADSL (Fig. 8B) suggesting that the drug effect may be mediated by multiple genes within a closely linked gene module.

All the drugs listed in Table 1 are either FDA approved or are currently undergoing clinical trial against various diseases, e.g., Nilotinib to treat chronic myeloid leukemia (89), Tirilazad against acute ischemic stroke (90), Ergotamine against migraine (91), etc. Extensive drug repurposing has been carried out recently to treat SC2 infection (77, 92) and corroborate our proposal. Nilotinib has been shown to be effective against covid-19 (93). Likewise, Temoporfin and Conivaptan have been shown to be effective against covid-19 based on *in silico* studies, where temoporfin targets 3C protease (94) and conivaptan targets viral non-structural protein 9 (Nsp9) (95). Phthalocyanine has been shown to be effective against preventing covid-19 in randomized trials (96). Bemcentinib has been shown to be effective against SC2 (97) and is being tested (trial NCT04890509) for efficacy in hospitalized covid-19 patients. Bemcentinib was designed for targeting AXL, a tyrosine kinase which signals via PI3K (98); however, our analysis identifies it as a lead molecule against ADSL and PBK. We use GeneMANIA software (99) to probe potential relationship between ADSL or PBK with AXL. As shown in Fig. 8A, there is a physical interaction between AXL and PBK via PIK3R2. Likewise, interaction can be seen among AXL and ADSL in Fig. 8B, suggesting that Bemcentinib’s effect may be mediated by multiple genes within a closely linked gene module.

## Discussion

We applied PathExt to investigate the response to SC2 infection in different cell lines (lung epithelium, bronchial epithelium and kidney cells), as well as in patient derived PBMCs. We also compared the response to SC2 with those for other respiratory viruses. Some of the key processes enriched among the PathExt-identified genes involve the immune system, cytokine signaling, metabolism, DNA replication and transcription, lipid mediated processes, etc. Our analysis revealed both similarity and dissimilarity in response to SC2 across different cell lines, reflecting different strategies evolved in different cell types likely governed by their specific regulatory networks. For instance, as A549 cell line, with low baseline ACE2 expression, has a lower viral load, its response, in terms of the enriched pathways among central TopNet genes, were different in comparison to Vero and Calu3 cell lines. Several previous studies have shown the links between viral load dynamics and disease severity. Viral load affects the host gene expression and downstream response pathways (66, 100). Blanco et al. have shown that only 0.1% viral reads were detected post SC2 infection in A549, whereas in Calu3 cell line, 15% of the reads were detected. They also observed different host transcriptional landscape in different cell lines based on virus replication rate post SC2 infection (16). Comparing the response to SC2 with those of other respiratory viruses (Influenza, SARS1, MERS, HPIV3 and RSV), we noted that the central TopNet genes are largely virus specific. However, the broad biological processes enriched among these genes exhibit a much greater degree of similarity across viruses, suggesting a virus-specific host response network that nevertheless affects common phenotypic response. Some common processes enriched among all the viruses include cytokine signaling, defense response, cell cycle regulation, inflammatory response, etc. However, processes like ATP metabolic process, carbohydrate derivative metabolic process, cardiomyocyte differentiation, phagocytosis, leukocyte activation and migration, etc. were found to be specific to SC2 response. Though activated and repressed TopNets shared very few central genes, they shared several processes enriched among the central genes, including cell cycle, phosphorylation, metabolism, etc., suggesting that these biological processes likely mediate both upregulation and downregulation of various genes in the global transcriptomic response to SC2 infection.

Conventional DEG-centered approaches can miss the genes which are not significantly differentially expressed but are nevertheless responsible for mediating, for instance, based on their post-translational modification state, the global transcriptional response in a given condition. PathExt addresses this limitation by focusing on genes that critically mediate significant path-level perturbations in the network. Thus, as expected, many of the central genes identified by PathExt exhibit much lower expression fold change relative to DEGs. Interestingly, some genes are identified as central in both activated as well as repressed TopNets (a feature unique to PathExt), consistent with pleiotropic function of genes. PathExt identified genes are largely disjoint from DEGs. In contrast to top PathExt genes, the top upregulated DEGs show significantly enriched pathways only in the Calu3 and Vero cell lines, with only one pathway enriched in the Vero cell line. The top downregulated DEGs exhibited no enrichment at all in any cell line.

Similarly in patient PBMC dataset, while the central genes both in activated TopNet as well as in upregulated DEGs were enriched for cell cycle and defense response, the downregulated DEGs did not show any enrichment while key genes in repressed TopNet were enriched for cytokine production. The pathways enriched among central genes in PBMC TopNets likely reflect both the innate endogenous response of immune cells to viral infection as well as response to systemic infection. However, a vast majority of central genes identified in PBMCs are specific to PBMCs, suggesting that transcriptional changes in PBMCs are largely in response to systemic infection. Demographic features were not statistically significant except “severity” category in Activated TopNet of PathExt. Central genes unique to ICU patients were associated with cell cycle processes whereas unique genes associated with non-ICU patients were enriched for cytokine mediated processes. Central genes in ICU patients exhibited a much greater homogeneity in their TopNet neighborhood across patients compared with central genes in non-ICU patients, suggesting that disease severity may be mediated by a more conserved genes and processes. Importantly, PathExt was substantially more effective in identifying potential anti-SC2 drug targets. PathExt-identified targets were enriched in 18% of the gold set comparisons, compared with only 5% (NULL expectation) for DEGs.

Previous studies have noted that genes with variable expression are more likely to be detected as differentially expressed in multiple contexts and do not reveal context-specific functional responses (9). Compared to reliance only on the differential expression, by exploiting the knowledge-based gene networks, and focusing on identifying key genes associated with significantly perturbed paths, PathExt represents a complementary, and in important ways, more effective approach. While previous works have exploited protein networks to infer transcriptomic perturbations, they have still relied on significantly differentially expressed genes and interpreted them in the context of the network (101, 102). We have previously demonstrated (12), superiority of PathExt over such integrative approaches that nevertheless rely on significant differential expression.

In summary, our work (1) further establishes the utility of PathExt, (2) provides a comparative analysis of key genes potentially driving the global transcriptomic response to SC2 and other respiratory viruses and across multiple cell lines and patient PBMCs, (3) identifies target genes validated in previously published benchmark anti-COVID-19 target gene datasets, far better than DEGs, (4) proposes novel targets against COVID-19, and (5) proposes FDA approved drugs or drugs in clinical trials, against the novel targets.

## Methods

### Data Collection and Processing

We collected 12 datasets from 2 studies, one published (16) and another unpublished (103), which include pre- and post-infection transcriptome in 5 different cell lines (NHBE, A549, Calu3, Vero, MRC5), each infected by one or more of the 6 viruses (SARS-CoV-2, SARS-CoV-1, MERS, RSV, HPIV3, and Influenza virus). In addition, we also obtained PBMC transcriptomes from 100 COVID-19 patients and 26 non-infected individuals as controls (54). In total, we had 13 datasets from 3 different studies, details of which are provided in Table 2. For each dataset (except Vero and PBMCs), we downloaded the raw reads using prefetch (104); most datasets were single end reads except Vero for which paired end reads were provided. Files were split using fastq-dump command (104) trimmed using Trim Galore-0.6.6 (105) at default parameters and the reads mapped to the human transcriptomic index version hg38 using SALMON v.1.12. (106). We proceeded only with those samples for which at least 60% of the reads were mapped. In the case of Vero cell line and patient PBMC data, we directly downloaded the TPM (transcripts per million) values provided from the GEO.

### Gene Expression Normalization and Node weight computation

For every cell line, mean gene expression was computed for the treated and control samples. Genes with TPM value 0 or greater than 100 were removed. For patient PBMC data, each case was analyzed individually and for the control, we took the mean expression of 26 control samples. Data was further filtered by removing the genes whose expression value was either 0 or above 100 in at least 50% of the PBMC case samples. Lastly, we also excluded the genes from the cell lines and PBMC data, which were not present in our human protein-protein interaction network. After the above filters, there were a total of 7,740 genes for cell lines (except Vero), 6,852 for Vero cell line. In case of patient PBMC datasets, the main dataset comprises 12,417 genes (Supplementary Table S18). All data was quantile normalized as done in previous studies (107, 108). Lastly, the normalized data was used to compute node weights which was then used to compute differentially expressed paths using PathExt software. Node (gene) weights to identify activated TopNet were computed as follows. While log(fold change) is reasonable choice for node weight, given the dependence of the magnitude of log(fold change) on the expression value, we instead computed the expected log(fold change) for a given control expression and then used the difference of observed and expected log(fold change) as the node weight. To compute the expected absolute log(fold change), we regressed absolute log(fold change) values across all genes against the gene expression in control samples using Loess fit, implemented in R (109). For activated TopNets the fold change was computed in cases relative to control, and for repressed TopNets the fold change was computed in control relative to cases. Computed node weights for Activated and Repressed TopNets in SC2 infected cell line (other than Vero) is provided in Supplementary Table S19, for Vero cell line is provided in Supplementary Table S20 and for the PBMCs is provided in the Supplementary Table S21. Node weight computed for remaining viruses is provided in Supplementary Table S22.

Given the node weights across samples, the PathExt tool computes the significant paths in two steps: (1) Top 0.1% shortest paths are selected and (2) Statistical significance of those selected paths is estimated based on data randomization and multiple testing corrected q-value threshold. We selected those percentiles and q-values which provide at least 300 nodes for a given TopNet. Once the TopNets were generated, we computed the ripple centrality score for each gene in the TopNet and top 100 central genes were selected for the further analysis. All the scripts used in this study are available in at https://github.com/hannenhalli-lab/covid19_project.

### GO enrichment analysis

Enriched pathways in a given TopNet (activated and repressed) were analyzed using the top 100 central genes using PANTHER software. Customized reference was used as a background during the enrichment analysis, where we considered only those genes which were used for the TopNet creation in this study. The reference was different for the Vero cell line, cell lines other than Vero and the patient PBMC data. ‘GO biological process complete’ was selected as Annotation Data Set, ‘Fisher’s Exact’ as Test Type, and ‘Calculate False Discovery Type’ as Correction method. As there could be redundant terms present in the result, we removed them by performing parent-child relation study using REVIGO software. The GO term and its corresponding FDR value was provided as an input with the resulting list option to be ‘Medium (0.7)’. Also, in the ‘Advanced options’, we selected ‘Yes’ in the remove obsolete GO terms, ‘Homo sapiens’ as the working species, and the ‘SimRel’ as semantic similarity measures. Finally, the circular plot representing the parent-child GO terms was created using the CirGo software (110). This tool requires the output of the REVIGO as an input. ‘GOSemSim’ package was used to compute the semantic similarity among the processes enriched in various cell lines, among different viruses and the patient PBMC data. In GOSemSim package, to calculate the similarities between two GO terms, we used Wang method which uses the topology of GO DAG graph structures to compute the semantic similarities. To combine two sets of GO terms, we utilized ‘rcmax’ method which considers the average of maximum similarity on each row and column on the matrix consisting of the similarities among two sets of GO terms. Heatmap plots for the similarity analysis are visualized using ggplot2 R package (111).

### Comparing most central genes across cell lines and viruses

Upset plot was generated by providing the list of genes of all the samples in a csv file format as an input to the server. Intervene server (112) at default parameters was used for generating the upset plots. For gene enrichment heatmap, we computed the Observed/Expected score. ‘pheatmap’ package in R was used for generating the heatmaps (113).

### Identification of novel drug targets and their potential inhibitors

First, we identified known drug targets of SC2 (till March 2021) from various published reports which includes CRISPR gene-knockout studies, viral-host protein-protein interactions (PPI) and experimentally screened drugs in various cell lines and animal models. These studies reported the host genes important for the viral replication and function. CRISPR-Cas studies provided the important therapeutic targets based on the gene knockdown studies and their downstream effects, whereas protein-protein interaction studies shed light on the targets physically interacting with the viral proteins. *In vitro* and *in vivo* studies in the cell lines and animal models provided the information about the genes which could be potentially targeted by different drugs. We excluded all these targets from our list of predicted targets. To provide specific recommendations for SC2, we also excluded the targets which were present in the TopNets (Activated & Repressed) for any other virus such as Influenza, HPIV3, RSV, SC1 and MERS. For other viruses, we removed all the genes present in the TopNets for the respective viral response in various cell lines. This left us with the targets unique to SC2 and we then prioritized the remaining central TopNet genes based on their frequency across cell line samples as novel potential drug targets. Similar approach was followed for the PBMC data.

Next, to propose potential inhibitors against our proposed novel targets, we performed a virtual screening process using Autodock Vina software (114). The 3D structures of the targets were downloaded from the RCSB-PDB (115) and further refined using Open Babel software (116). Active site information of the protein molecules was computed using P2RANK software (117). Next, a drug library was created for the virtual screening process, where we considered only those drug molecules which are either FDA approved or are under clinical trials; SMILES formatted files of these drugs were downloaded from the ZINC database (118) and were further converted to MOL2 file format using openbabel. The ligand and the receptor files were prepared in the ‘pdbqt’ file format required by vina for the docking purpose. The center and the grid size of the receptor molecules was computed using UCSF Chimera software (119), based on the P2RANK software active site result. Lastly, based on the Autodock Vina affinity score and Root Mean Square Deviation (RMSD) value, we proposed the drug molecules which could potentially inhibit the function of these new targets.

## Supplementary Material

Supplement 1

1

## Figures and Tables

**Table 1:** Proposed small molecules potentially inhibiting the novel anti-SC2 targets.

Sr No.	Target	Proposed Drug Molecules
**Cell Line**		
1	ADCY2	Phthalocyanine, Tirilazad, Temoporfin, Telcagepant, Laniquidar
2	ADSL	Nilotinib, Lixivaptan, Telcagepant, Doramapimod, Bemcentinib
5	OCRL	Conivaptan, Implitapide, Ergotamine, Fluspirilene, Tolvaptan
7	TIAM1	Cipargamin, MK-3207, Adozelesin, Hypericin, Tariquidar
**PBMC**		
8	BUB1	Hypericin, Erismodegib, Hemin, Irinotecan, TMC-647055
10	PBK	Conivaptan, Bemcentinib, Dihydroergotamine, Phthalocyanine, UK-432097

**Table 2:** Summary of datasets used in the study

Dataset Name	Description	Sample Size	Platform	Reference
SC2^1^_Vero	SC2 infected African green monkey kidney epithelial cell line	6	Bulk RNASeq	Riva et.al.
SC2_Calu3	SC2 infected human lung adenocarcinoma cell line	6	Bulk RNASeq	Blanco et.al.
SC2_NHBE	SC2 infected primary normal human bronchial epithelium cell line	6	Bulk RNASeq	Blanco et.al.
SC2_A549	SC2 infected human alveolar basal epithelial cells	6	Bulk RNASeq	Blanco et.al.
SC2_A549_ACE2	SC2 infected human alveolar basal epithelial cells with higher ACE2 expression	6	Bulk RNASeq	Blanco et.al.
INF^2^_A549	Influenza infected human alveolar basal epithelial cells	6	Bulk RNASeq	Blanco et.al.
INF_NHBE	Influenza infected primary normal human bronchial epithelium cell line	6	Bulk RNASeq	Blanco et.al.
INF_DNS1_NHBE	Influenza (lacking gene NS1) infected primary normal human bronchial epithelium cell line	6	Bulk RNASeq	Blanco et.al.
HPIV3^3^_A549	HPIV3 infected human alveolar basal epithelial cells	6	Bulk RNASeq	Blanco et.al.
RSV^4^_A549	RSV infected human alveolar basal epithelial cells	4	Bulk RNASeq	Blanco et.al.
SC1^5^_MRC5	SC1 infected diploid cell culture line composed of fibroblast	6	Bulk RNASeq	GSE56192
MERS^6^_MRC5	MERS infected diploid cell culture line composed of fibroblast	6	Bulk RNASeq	GSE56192
SC2_PBMCs	SC2 infected peripheral blood mononuclear cells	126	Bulk RNASeq	Overmyer et.al.

1: SARS-CoV-2;

2: Influenza;

3: Human Parainfluenza Virus;

4: Respiratory Synctial Virus;

5: SARS-CoV-1;

6: Middle East Respiratory Syndrome
