# Supplementary material for: A path-based analysis of infected cell line and COVID-19 patient transcriptome reveals novel potential targets and drugs against SARS-CoV-2
Source: Res Sq. 2022 Mar 21:rs.3.rs-1474136. Preprint. [Version 1] doi: 10.21203/rs.3.rs-1474136/v1 (PMC9013431; doi:10.21203/rs.3.rs-1474136/v1)
Supplement: 1 [file NIHPPrs1474136v1-supplement-1.pdf]

Supplementary Figures

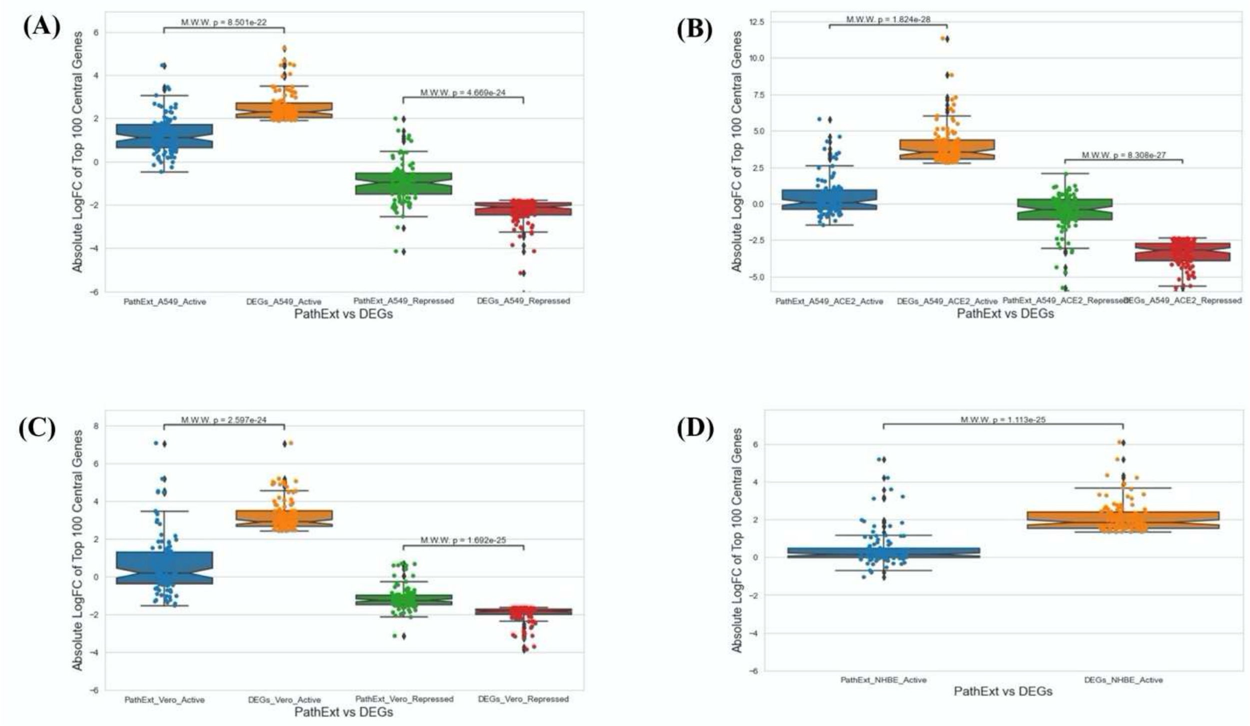

Supplementary Figure S1. Fold Change comparison of the top 100 frequent genes of PathExt TopNets and DEGs in various SC2 infected cell lines in (A) A549 cell line; (B) A549_ACE2 cell line; (iii) Vero Cell line; and (iv) NHBE cell line.

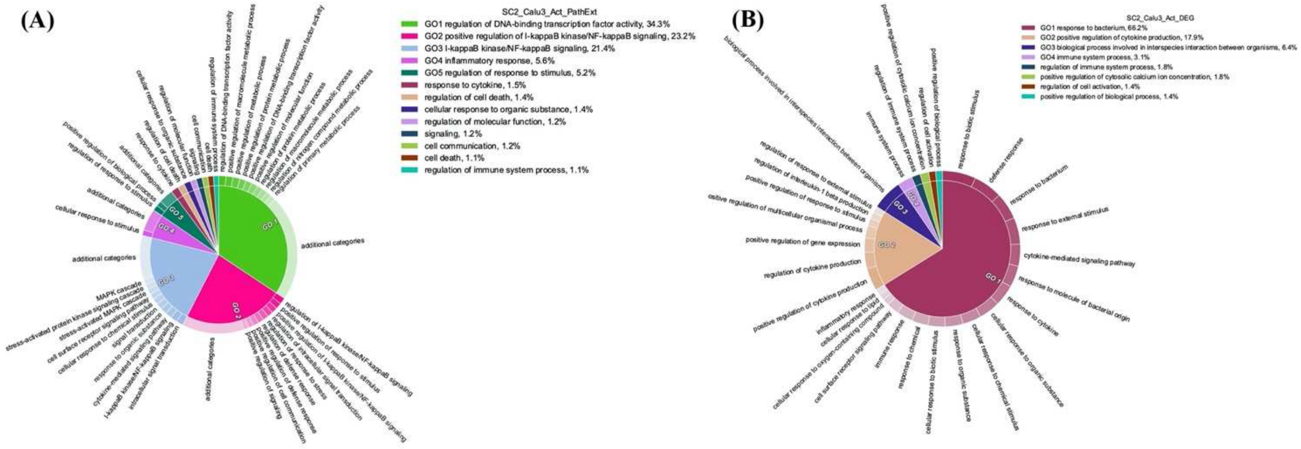

Supplementary Figure S2: Parent and child enriched pathways associated with upregulated DEGs in SC2 infected Calu3 cell line. Most frequent top 100 upregulated genes were obtained across patient PBMC data. Enriched biological processes was obtained by performing Gene Ontology study followed by parent child relationship, shown in the form of circular visualization plot.

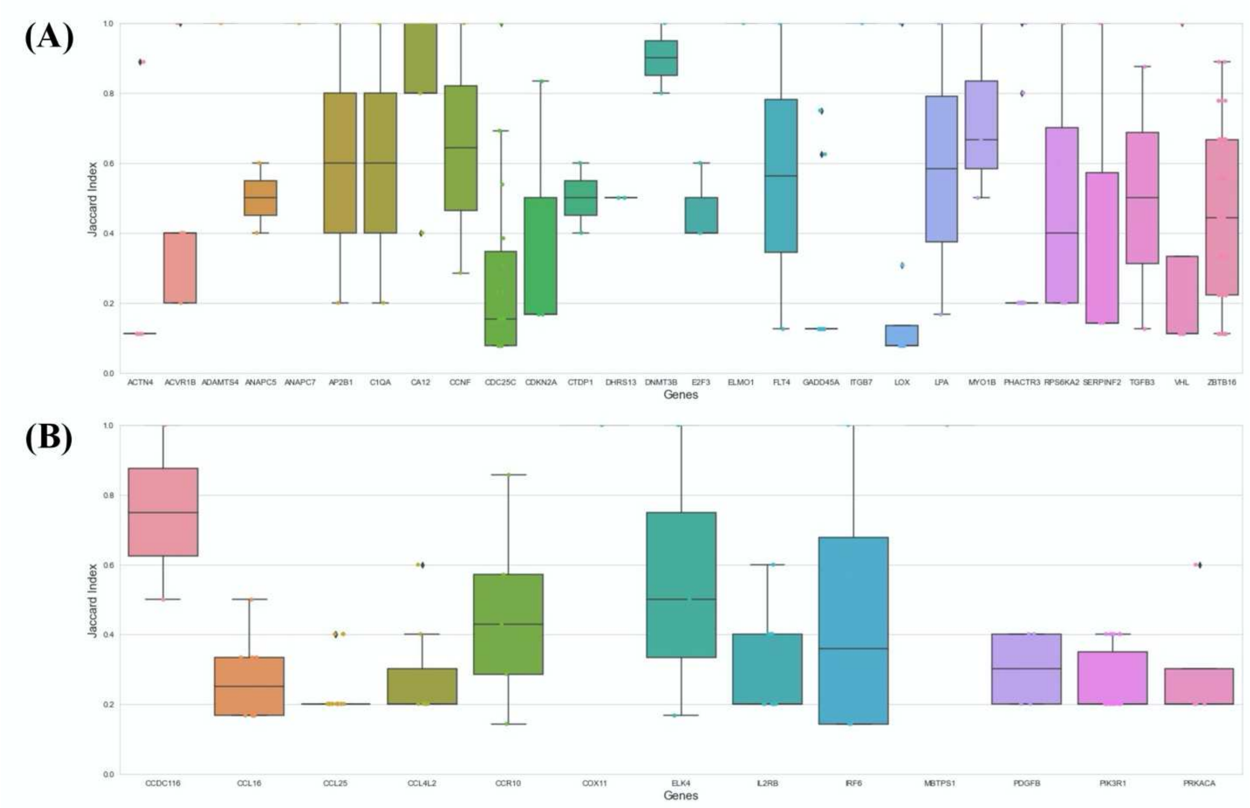

Supplementary Figure S3: Cross-sample overlap on TopNet neighbors of central genes. For each central genes (x-axis), the figures show the distribution of Jaccard Index of all sample-pair overlap between the TopNet neighbors of the central gene, for Activated TopNet for ICU patients (A) and non-ICU patients (B).

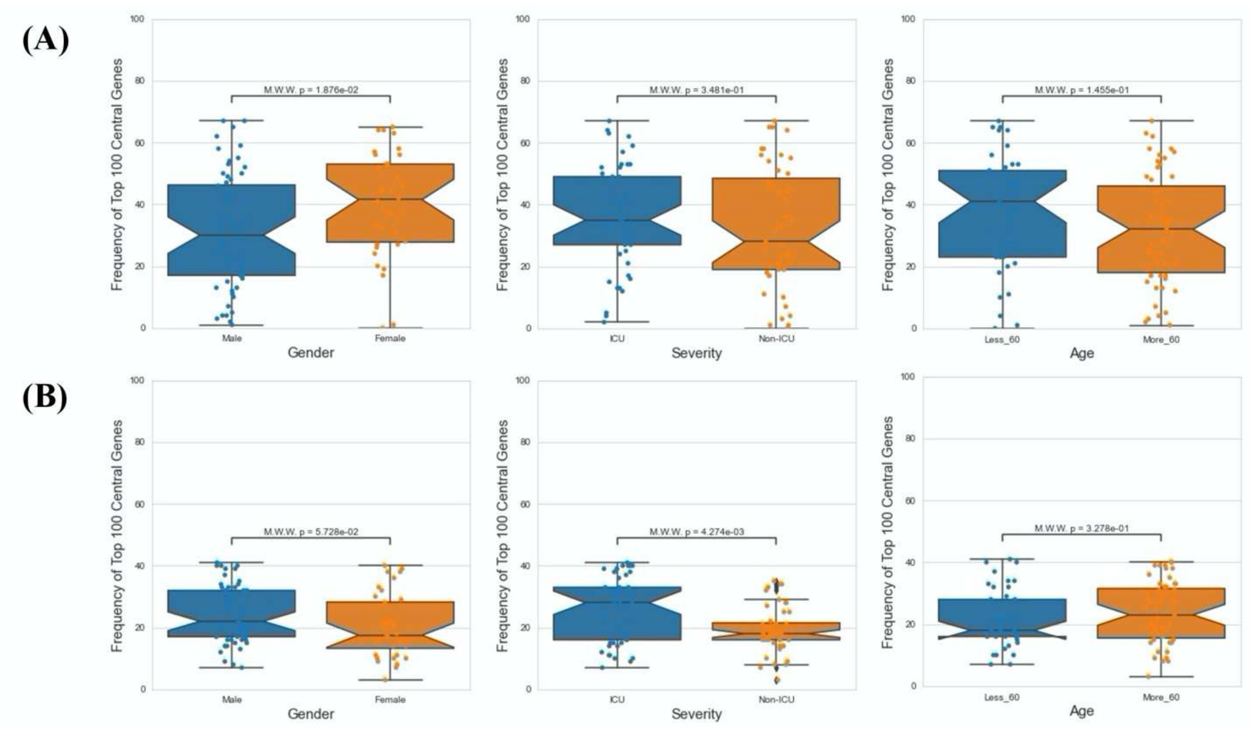

Supplementary Figure S4: Demographic features analysis. Mann Whitney Test was performed to check statistical significance between top 100 differentially expressed genes and various demographic features (age, sex and severity). In case of upregulated DEGs, "Gender" was found to be the only statistically group among activated TopNet genes (A) and in case of downregulated DEGs, "Severity" was found to be the only statistically group among repressed TopNet genes (B).

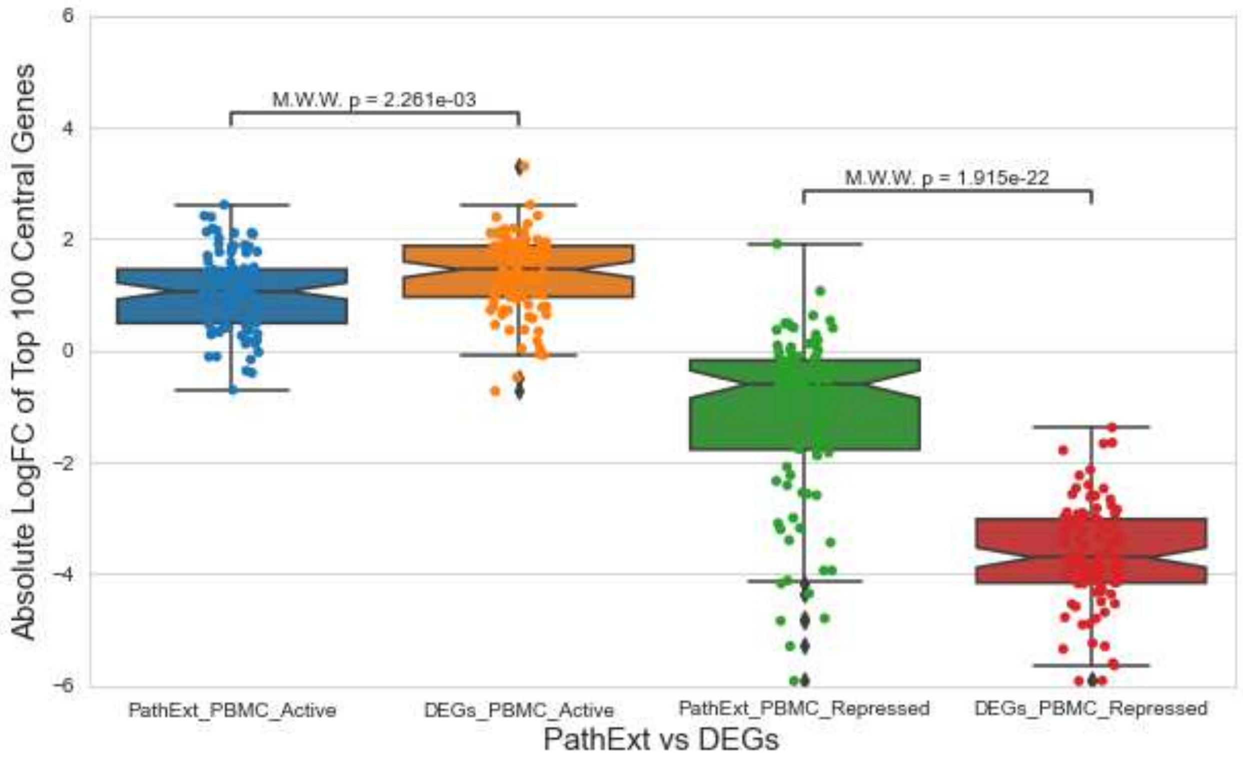

Supplementary Figure S5. Fold Change comparison of the top 100 frequent genes of PathExt TopNets and DEGs. LogFC comparison of the top 100 genes between PathExt and DEGs obtained from the SC2-infected patient PBMC data. Differential expression is estimated in infected relative to uninfected cells. Mean expression of the genes across 100 patients were considered while plotting.

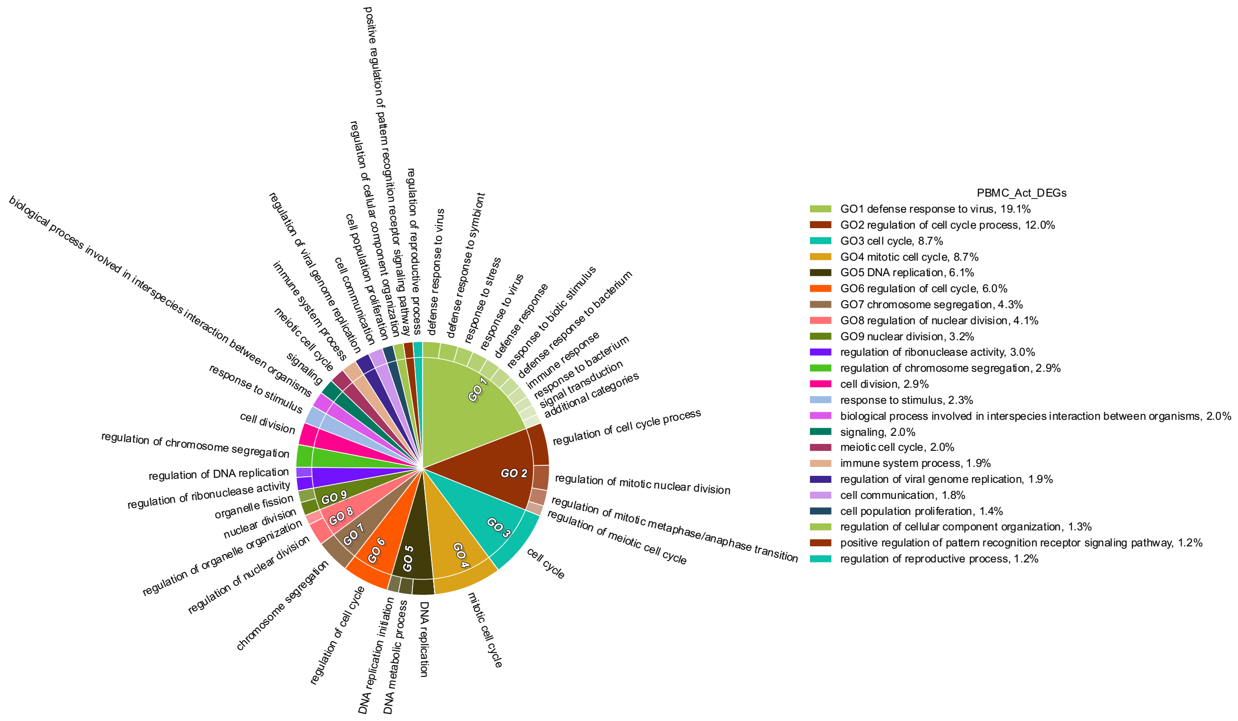

Supplementary Figure S6. Parent and child enriched pathways associated with upregulated DEGs in patient PBMC. Most frequent top 100 upregulated genes were obtained across patient PBMC data. Enriched biological processes was obtained by performing Gene Ontology study followed by parent child relationship, shown in the form of circular visualization plot.

Supplementary Files

This is a list of supplementary files associated with this preprint. Click to download.

- SupplementaryV5.xlsx
